# Editorial: Novel signaling pathways and therapy in breast cancer

**DOI:** 10.3389/fonc.2023.1215023

**Published:** 2023-05-16

**Authors:** Eriko Katsuta, Mateusz Opyrchal

**Affiliations:** ^1^ Department of Cell Stress Biology, Roswell Park Comprehensive Cancer Center, Buffalo, NY, United States; ^2^ Department of Oncology, Yokohama City University, Graduate School of Medicine, Yokohama, Japan; ^3^ Indiana University School of Medicine, Department of Medicine, Division of Hematology/Oncology, Indianapolis, IN, United States

**Keywords:** breast cancer, signaling pathway, molecular target, novel therapy, metastasis

Breast cancer is the most common malignancy in women worldwide. Advances in breast cancer prevention and treatment have achieved a 90% five-year overall survival rate (1). However, a large number of patients with breast cancers recur over time, sometimes many decades after the initial diagnosis. Although there are multiple treatment options for breast cancer including endocrine therapy, chemotherapy, molecular targeted therapy, radiotherapy, and immunotherapy, there are still more than 40,000 breast cancer-related deaths annually in the US. Therefore, investigating molecular mechanisms of tumor progression, resistance and metastasis are crucial to better understand and identify at-risk patients and develop novel targets and treatment strategies in order to improve breast cancer patient prognosis and outcomes.

The goals of this Research Topic were to highlight research into the molecular mechanisms underlying cancer progression in patients with breast cancer. This Research Topic aimed to focus on identifying novel intrinsic and extrinsic molecular pathways. We emphasized research with potential clinical applications either identifying at-risk patients for failing standard therapy approaches or targeting novel signaling pathways with a promise of novel therapeutics.

There are two basic research studies that focused on transcription factor E2F family. One study, Zhang explored the role of polymerase delta 2 (POLD2) in triple-negative breast cancer (TNBC). They found a high expression of POLD2 in TNBC compared to normal tissue as well as other subtypes of breast cancer. Further, high expression of POLD2 was associated with poor prognosis in TNBC patients. This finding was supported by experimental data showing that knockdown of POLD2 inhibited cell proliferation in TNBC cells. As a mechanism, they elucidated that a transcription factor, E2F1, regulates POLD2 expression by directly binding to the POLD2 promoter. A second study, Zheng et al. examined E2F8 role in a basal-like subtype of breast cancer by bioinformatical and experimental approaches. They found that E2F8 expression was associated with poor prognosis in a basal-like breast cancer. E2F8 was associated with dysregulated cell cycle and inhibition of apoptosis. Its expression was also correlated with higher chemotherapy sensitivity. Interestingly, it was also associated with higher CD4+ and CD8+ T cell infiltration observed in the tumors.

There are two translational studies focusing on biomarker discovery and chemo-sensitivity. Zhou et al. investigated the role of high mobility group protein B3 (HMGB3) expression in breast cancer. They found that HMGB3 was highly expressed in breast cancer compared to normal tissue and its expression was associated with an aggressive phenotype and chemo-resistance in both breast cancer cell line and patient samples. They suggested that HMGB3 might be a potential biomarker not only for prognosis, but also for detecting and making treatment decisions in breast cancer. Another study, Qian et al. focused on the impact of Integrin α6β4 on chemotherapy in TNBC. Integrin α6β4 is highly expressed and associated with aggressive phenotype in TNBC. They also found that expression of integrin α6β4 sensitized breast cancer cells to platinum agents, including cisplatin and carboplatin, through activation of DNA damage response pathway.

Lasty, there is a clinical study. Chen et al. examined the efficacy and safety of anlotinib, an oral multi kinase inhibitor targeting vascular endothelial growth factor receptor (VEGFR), fibroblast growth factor receptor (FGFR), platelet-derived growth factor receptors (PDGFR), and c-kit. It is a retrospective study of 56 metastatic breast cancer patients who failed standard treatment. Although the study is not controlled and offers a retrospective look at the institutional experience the findings show that anlotinib-based treatment was relatively well tolerated with some promising responses. Targeting VEGF in breast cancer has been a source of ongoing debate and although limited by the design, the study adds to the overall knowledge and might lead to novel proposals to test strategies under more rigorous conditions.

Despite recent progress in the area of oncology and specifically in the treatment of breast cancer, there remains a significant number of patients who will die from their disease. Identifying novel signaling pathways in order to understand the process of metastasis and resistance to current treatment continues to be the key to helping more patients. Another important area of research is identifying patients whose breast cancer has inherent resistance to current standard of care therapies. These patients should be preferentially steered toward treatment in clinical trials testing novel approaches. Discovering novel signaling pathways will shed more light on biology of breast cancer and allow for improved prognosis, patient selection for specific treatments and novel therapies.

## Author contributions

All authors have made a substantial and direct contribution to the work and approved it for publication.
